# A comparative study on the clinical differences in traditional Chinese medicine pattern in ulcerative colitis utilizing multidimensional data

**DOI:** 10.3389/fmed.2026.1830908

**Published:** 2026-07-15

**Authors:** Xingyao Lu, Yichuan Xv, Enjia Guo, Hongyi Hu, Dongya Chen, Chan Lv

**Affiliations:** 1 Department of Gastroenterology and Hepatology, Hangzhou Red Cross Hospital, Hangzhou, China; 2 Department of Gastroenterology, Longhua Hospital Affiliated to Shanghai University of Traditional Chinese Medicine, Shanghai, China; 3 Department of Gastroenterology, Shuguang Hospital Affiliated to Shanghai University of Traditional Chinese Medicine, Shanghai, China

**Keywords:** biomarker, endoscope, histopathology, traditional Chinese medicine, ulcerative colitis

## Abstract

**Background:**

Ulcerative Colitis (UC) is a chronic autoimmune disease with increasing incidence, particularly in Asia. Traditional Chinese Medicine (TCM) provides effective treatment through pattern differentiation. This study integrated multidimensional data to investigate the clinical differences between two common TCM patterns, dampness-heat in the large intestine (Da-Chang-Shi-Re, DCSR) and spleen deficiency with dampness accumulation (Pi-Xu-Shi-Yun, PXSY), providing a basis for revealing the biological connotation of TCM patterns.

**Methods:**

A total of 180 UC patients (90 with DCSR pattern and 90 with PXSY pattern) were enrolled from March 2024 to March 2025. Peripheral inflammatory markers were measured. Endoscopic features were assessed using four scoring systems, along with detailed endoscopic characteristics. Multivariate regression models were constructed, adjusting for confounding variables including sex, medication history, baseline lesion severity, disease duration and smoking status. Intestinal histological injury was quantified using the Robarts Histopathology Index (RHI). Immunohistochemical staining was performed to assess tissue CD4 and myeloperoxidase (MPO) expression. Immunofluorescence staining was further applied to detect the expression of NADPH oxidase 2 (NOX2), CD11b, and citrullinated histone H3-labelled neutrophil extracellular traps (NETs).

**Results:**

After adjusting for multiple covariates, patients with DCSR pattern exhibited significantly higher levels of neutrophil-to-lymphocyte ratio (*β* = 1.602, adjusted-*p* < 0.006), platelet-to-lymphocyte ratio (*β* = 75.851, adjusted-*p* < 0.006), and neutrophil-to-albumin ratio (*β* = 0.044, adjusted-*p* < 0.006) compared to patients with PXSY pattern. Endoscopically, DCSR patients showed more severe inflammation with higher endoscopic scores. After adjusting for confounding factors, Multivariate analysis showed that ulcer formation was an independent differentiating factor between the two (OR = 2.429, 95% CI: 1.209–4.880, *p* = 0.013). Histologically, the DCSR pattern demonstrated more severe mucosal damage with significantly higher RHI scores and increased MPO^+^ cells (*p* < 0.01). In addition, patients in the DCSR group showed higher expression levels of NOX2, CD11b, and NETs in the colon.

**Conclusion:**

The DCSR pattern is characterized by more severe systemic and local inflammation, while the PXSY pattern reflects a milder, chronic inflammatory state. Neutrophil activation was also markedly elevated among patients presenting the DCSR pattern. The integration of multidimensional biomarkers provides objective evidence for TCM pattern differentiation and offers valuable insights for personalized treatment strategies in UC management.

## 1 Introduction

Ulcerative Colitis (UC) is a chronic relapsing autoimmune disease, and its incidence has been steadily increasing worldwide, particularly in Asia (1). Traditional Chinese Medicine (TCM) is increasingly utilized in UC management, with its definite therapeutic efficacy proved by numerous clinical and experimental studies (2–4). According to classical TCM theory, UC can be classified into several patterns, including dampness-heat in the large intestine (Da-Chang-Shi-Re, DCSR), spleen deficiency and dampness accumulation (Pi-Xu-Shi-Yun, PXSY), cold-heat complex, and liver depression with spleen deficiency, among which DCSR and PXSY are the most common patterns. TCM pattern differentiation is conducted through the comprehensive analysis of clinical symptoms, tongue appearance, and pulse characteristics. Although distinct differences exist between various pattern types from TCM theory, the underlying biological disparities remain unclear, which, to some extent, limits the modernization and standardization of TCM.

Using modern technology to study the biological differences between patterns can provide evidence-based support for TCM pattern differentiation and treatment. Emerging studies have explored the differentiation of TCM patterns using microbiome, metabolomics and peripheral blood parameters (5–7). Zhang et al. found that different TCM patterns exhibit distinct gut-microbiota profiles. Specifically, patients with the DCSR pattern exhibited a higher relative abundance of the *Streptococcus* genus, whereas those with the PXSY pattern showed increased levels of *Lachnoclostridium* (5). Lian et al. (6) applied metabolomic analysis and found that UC patients with spleen-qi deficiency may be associated with perturbations in lipid metabolism. However, these studies are insufficient for comprehensively describing the clinical characteristics of different TCM patterns. Therefore, this study aims to systematically investigate the clinical differences between two classic TCM patterns in UC, namely DCSR and PXSY, by integrating peripheral inflammatory markers, endoscopic features, and histological characteristics. These findings are expected to provide data-driven support for the objectification and standardization of TCM pattern differentiation.

## 2 Methods

### 2.1 Study population

A total of 180 UC patients were enrolled from both outpatient and inpatient settings between March 2024 and March 2025, of whom 90 were diagnosed with the DCSR pattern and 90 were diagnosed with the PXSY pattern. All participants were recruited from Longhua Hospital affiliated with Shanghai University of Traditional Chinese Medicine. This study has been approved by the Ethics Committee of Longhua Hospital (Ethics Approval No.: 2024LCSY024) and has been registered in the International Traditional Medicine Clinical Trial Registry (website: https://itmctr.ccebtcm.org.cn/, Clinical Research Registration: ITMCTR2025002385).

### 2.2 Sample size calculation

A total of 16 candidate variables were included in this study. We hypothesized that 7 to 10 of these variables were associated with TCM patterns. Following the widely accepted 10-events-per-variable rule for sample size estimation in regression analyses (8), we calculated the required sample size for each TCM pattern group to be 70–100 participants. As the allocation ratio between the two TCM pattern groups was set to 1:1, the total required sample size was 140–200 participants. Ultimately, 180 patients were enrolled in the study, with 90 participants allocated to each group.

### 2.3 Inclusion and exclusion criteria

*Inclusion criteria*: (a) The diagnosis of UC is made based on a comprehensive assessment that includes clinical symptoms, endoscopic findings, histological examination, and biological markers (9). (b) The diagnosis of TCM pattern must include at least two of the following primary and secondary symptoms (10). The main symptoms of the DCSR pattern include diarrhea, mucus-pus-blood stools, abdominal pain, and urgency with a feeling of incomplete evacuation, while the secondary symptoms include anal burning, abdominal distension, short and dark urination, and dry mouth with a bitter taste. The main symptoms of the PXSY pattern include abundant mucus with little blood, loose stools accompanied by undigested food, and abdominal fullness, while the secondary symptoms include dull abdominal pain, fatigue, poor appetite, listlessness and reluctance to speak. (c) The interval between colonoscopy and laboratory tests and TCM diagnosis should be less than 48 h. (d) Patients aged 18 years or older, regardless of gender. (e) Patients who voluntarily consent to participate in this study.

*Exclusion criteria*: (a) Patients with severe comorbid systemic diseases or serious infections. (b) Patients with severe underlying conditions affecting the heart, brain, liver, or kidneys. (c) Pregnant or lactating women. (d) Patients with a history of significant psychiatric disorders who are currently undergoing treatment.

### 2.4 TCM pattern diagnosis

The diagnosis of TCM patterns was independently made by two physicians both expertise in gastroenterology and TCM. Physicians were blinded to patient identifiers, endoscopic findings, histopathology reports, and laboratory data. Diagnoses were rendered according to the criteria specified in the Experts Consensus on Traditional Chinese Medicine Diagnosis and Treatment of Ulcerative Colitis (10). Cases with discordant diagnoses were excluded from the final analysis.

### 2.5 Data collection

Baseline demographic information, including gender, age, height, and weight, was collected for each participant. The following laboratory parameters were collected: neutrophil (NEU), platelet (PLT), monocyte (MONO), and lymphocyte (LYM) counts, albumin (ALB) and C-reactive protein (CRP) levels, and erythrocyte sedimentation rates (ESR). Colonoscopy findings for inpatients were defined as those obtained from the first colonoscopy conducted following hospital admission. The extent of intestinal lesions was categorized based on the Montreal classification into proctitis (E1), left-sided colitis (E2), and extensive colitis (E3). Four scoring systems were used to assess endoscopic disease severity, including the Mayo Endoscopic Score (MES), Modified Mayo Endoscopic Score (MMES), Ulcerative Colitis Endoscopic Index of Severity (UCEIS), and Ulcerative Colitis Clinical Index of Severity (UCCIS). The endoscopic evaluation of the disease was performed by two experienced endoscopists, with a third independent reviewer involved in case of discrepancies between the two. All patients undergoing endoscopy have biopsies taken, which are then fixed, dehydrated, sliced, and stained with hematoxylin and eosin (HE) for evaluation by experienced pathologists. Histological inflammation was quantified using the Robarts Histopathology Index (RHI), which includes four components, and the total score is calculated based on these components (11, 12). The endoscopists and pathologists remain unaware of the TCM patterns and clinical history of the patients.

### 2.6 Immunohistochemistry

Immunohistochemistry (IHC) was performed on paraffin-embedded sections of colonic tissue from DCSR pattern (*n* = 25) and PXSY pattern (*n* = 25). Following deparaffinization in xylene and rehydration through graded ethanol, antigen retrieval was carried out by heating the sections in citrate buffer (10 mM, pH 6.0) at 95 °C for 20 min. Endogenous peroxidase activity was quenched with 3% hydrogen peroxide in methanol for 20 min at room temperature. To block non-specific binding, sections were incubated with 5% normal goat serum in phosphate-buffered saline for 1 h at room temperature, The sections were then incubated overnight at 4 °C with primary antibodies against CD4 (1:5000, HA723511, HUABIO) and myeloperoxidase (MPO) (1:1000, HA721146, HUABIO). The following day, secondary antibodies were applied, and color development was achieved using 3,3′-diaminobenzidine (DAB). After dehydration and mounting, the slides were observed under a microscope, and images were taken at 200× and 400× magnification. Image J software was used to quantitatively analyze the positively stained areas under high magnification for each slide. Three representative field of view was selected from each slide for statistical analysis. Researchers performing immunohistochemical experiments and quantitative analyses were unaware of the patient’s clinical information.

### 2.7 Tyramide signal amplification immunofluorescence

Tyramide signal amplification (TSA) immunofluorescence was employed to detect the expression and colocalization of target proteins in colonic tissue sections. In brief, paraffin-embedded sections were dewaxed in xylene, rehydrated through a graded ethanol series, and subjected to antigen retrieval by microwave heating in pH 6.0 citrate buffer for 20 min. After cooling to room temperature, sections were washed with PBS and incubated with 3% H_2_O_2_ to quench endogenous peroxidase activity. Non-specific binding was blocked with 5% normal goat serum for 30 min at room temperature. The slides were incubated overnight at 4 °C with primary antibodies. In this section, we used four primary antibodies, including those against citH3 (1:100, P63763-1B1, Abmart), CD11b (1:200, T55019, Abmart), MPO (1:1000, HA721146, HUABIO), and NOX2 (1:400, TD6520, Abmart). Following three washes with PBST, sections were incubated with HRP-conjugated secondary antibody for 1 h at room temperature. After additional PBST washes, freshly prepared TSA working solution was applied and incubated for 10 min in the dark at room temperature. For double immunofluorescence labeling, the antigen retrieval, blocking, primary antibody incubation, HRP-secondary antibody incubation, and TSA development steps were repeated using a second fluorophore. Finally, sections were mounted with an anti-fade mounting medium containing DAPI and imaged using a fluorescence scanner. Three representative fields of view were selected from each slide for statistical analysis. Quantitative analysis was performed using ImageJ software. The researchers performing the immunofluorescence were unaware of the patients’ clinical information.

### 2.8 Statistical analysis

Data were analyzed using R version 4.3.3. Continuous variables were presented as mean ± SD or median (IQR), while categorical variables were expressed as n (%). For non-normally distributed data, the Mann–Whitney *U* test was used for intergroup comparisons. Chi-square analysis was conducted for categorical data. To assess inter-rater reliability, we calculated Cohen’s Kappa coefficient. Regression analysis was performed to assess the association between laboratory indications and endoscopic features and TCM pattern. In the multivariate regression analysis, gender, medication history, extent of intestinal lesions, disease course, and smoking status were used as covariates. The Benjamini–Hochberg (BH) method was applied to correct for multiple comparisons. *p* < 0.05 was set to indicate statistical significance in all analyses.

## 3 Results

### 3.1 General characteristics

This study included a total of 180 participants (Table 1), comprising 90 patients with the DCSR pattern and 90 patients with the PXSY pattern. No significant differences were observed between the two groups in terms of gender, age, or body mass index (BMI). In terms of disease characteristics, patients with the DCSR pattern exhibited more severe disease activity and a broader extent of lesions. Specifically, the median Mayo score was 9 (IQR: 7–10) for the DCSR group, compared with 5 (IQR: 4–6) for the PXSY group. The majority of DCSR patients presented with moderate (67.78%) or severe (22.22%) active disease, with extensive colitis being the most common lesion type (54.44%). In contrast, in the PXSY group, mild (63.33%) or moderate (36.67%) disease severity was more common.

**Table 1:** General information of UC participants.

Variables	DCSR (*n* = 90)	PXSY (*n* = 90)	*p*
Sex, *n* (%)			
Male	53 (58.89)	51 (56.67)	0.763
Female	37 (41.11)	39 (43.33)	
Age, mean ± SD	46.04 ± 15.09	45.72 ± 14.08	0.882
BMI, M (Q₁, Q_3_)	22.07 (19.43, 24.77)	22.65 (20.43, 24.92)	0.317
NEU, M (Q₁, Q_3_)	5.54 (4.32, 6.90)	3.99 (3.47, 4.95)	<0.001
PLT, M (Q₁, Q_3_)	290.00 (241.25, 364.50)	242.00 (200.75, 276.00)	<0.001
LYM, M (Q₁, Q_3_)	1.42 (1.21, 1.78)	1.68 (1.44, 2.04)	<0.001
MONO, M (Q₁, Q_3_)	0.53 (0.39, 0.67)	0.47 (0.39, 0.62)	0.238
ALB, M (Q₁, Q_3_)	38.45 (34.95, 40.85)	40.80 (39.10, 41.90)	<0.001
CRP, M (Q₁, Q_3_)	3.83 (1.02, 15.89)	1.07 (0.50, 3.66)	<0.001
ESR, M (Q₁, Q_3_)	28.50 (13.25, 45.00)	18.00 (8.25, 29.00)	0.001
Mayo, M (Q₁, Q_3_)	9.00 (7.00, 10.00)	5.00 (4.00, 6.00)	<0.001
Disease Course, M (Q₁, Q_3_)	4.50 (2.00, 10.00)	5.00 (2.00, 12.00)	0.416
Smoking status			
Yes	78 (86.67)	77 (85.56)	0.829
No	12 (13.33)	13 (14.44)	
Montreal, *n* (%)			
E1	6 (6.67)	23 (25.56)	<0.001
E2	35 (38.89)	39 (43.33)	
E3	49 (54.44)	28 (31.11)	
Severity, *n* (%)			
Mild	9 (10.00)	57 (63.33)	<0.001
Moderate	61 (67.78)	33 (36.67)	
Severe	20 (22.22)	0 (0.00)	
Treatment			
None	14 (15.56)	11 (12.22)	0.640
5-Aminosalicylic Acid	41 (45.56)	42 (46.67)	
TCM	13 (14.44)	8 (8.89)	
5-Aminosalicylic acid+TCM	15 (16.67)	20 (22.22)	
Biologics	7 (7.78)	9 (10.00)	

### 3.2 The difference of laboratory indications between two TCM patterns

Significant differences in peripheral inflammatory markers were observed between the DCSR and PXSY groups. As shown in Table 1, patients in the DCSR group exhibited significantly higher levels of NEU, PLT, CRP and ESR compared to the PXSY pattern (*p* < 0.05). There were no differences in MONO levels between the two groups (*p* = 0.238). Additionally, DCSR pattern exhibited lower levels of LYM and ALB than those in the PXSY pattern (*p* < 0.05).

In recent years, several novel inflammatory markers have been identified, such as the neutrophil-to-lymphocyte ratio (NLR), platelet-to-lymphocyte ratio (PLR), lymphocyte-to-monocyte ratio (LMR), and neutrophil-to-albumin ratio (NAR), which offer greater specificity and sensitivity in assessing the severity of UC (13, 14). Accordingly, we compared NLR, NAR, PLR, and LMR between the two TCM patterns using linear regression analysis (Table 2). DCSR group exhibited significantly elevated levels of CRP, ESR, NLR, PLR, and NAR, while the LMR was significantly reduced (*p* < 0.05). After adjusting for gender, medication history, extent of intestinal lesions, disease duration, and smoking status, patients in the DCSR group still showed higher NLR (*β* = 1.602, *p* < 0.001, 95% CI: 0.991 ~ 2.214), PLR (*β* = 75.851, *p* < 0.001, 95% CI: 48.440 ~ 103.262), NAR (*β* = 0.044, *p* < 0.001, 95% CI: 0.028 ~ 0.060) levels, but lower LMR (*β* = −0.423, *p* = 0.041, 95% CI: −0.829 ~ −0.017) levels, compared to the PXSY group. After following BH correction, the significance of NLR, PLR, and NAR persisted (adjusted-*p* < 0.05) but not the LMR. These results indicate a significant difference in peripheral inflammatory burden between the two TCM patterns. Furthermore, we noted a close correlation between the differentially expressed inflammatory markers and NEUs (NLR and NAR), suggesting their potential involvement in the distinct clinical characteristics of the two patterns.

**Table 2:** Linear regression analysis of laboratory parameters between different TCM patterns.

Variable	Unadjusted model				Adjusted model^*^				Adjusted-*p*
	*β*	SE	*P*	95% Cl	*β*	SE	*P*	95% Cl	
CRP	8.771	3.658	0.018	1.552, 15.991	6.320	3.792	0.097	−1.165, 13.805	0.116
ESR	9.172	3.841	0.018	1.592, 16.751	5.745	4.003	0.153	−2.157, 13.648	0.153
NLR	1.821	0.292	<0.001	1.245, 2.397	1.602	0.310	<0.001	0.991,2.214	<0.006
PLR	81.419	13.033	<0.001	55.699, 107.138	75.851	13.886	<0.001	48.440, 103.262	<0.006
LMR	−0.549	−0.196	0.006	−0.935, −0.162	−0.423	0.206	0.041	−0.829, −0.017	0.0615
NAR	0.052	0.008	<0.001	0.037, 0.067	0.044	0.008	<0.001	0.028, 0.060	<0.006

*Adjusted confounders: gender, medication history, extent of intestinal lesions, disease duration, and smoking status.

### 3.3 The difference of endoscopic features between two TCM patterns

Endoscopic features have been recognized as the most accurate method for diagnosing, assessing, monitoring, and predicting recurrence in UC. The MES is extensively utilized in clinical practice, while the UCEIS has shown superior predictive ability in the need for treatment escalation (15–17). Modified scoring systems, such as the UCCIS and MMES, offer the advantage of evaluating variations in inflammatory burden across different intestinal segments, but with relatively complex calculation processes (18, 19).

**Figure 1:**
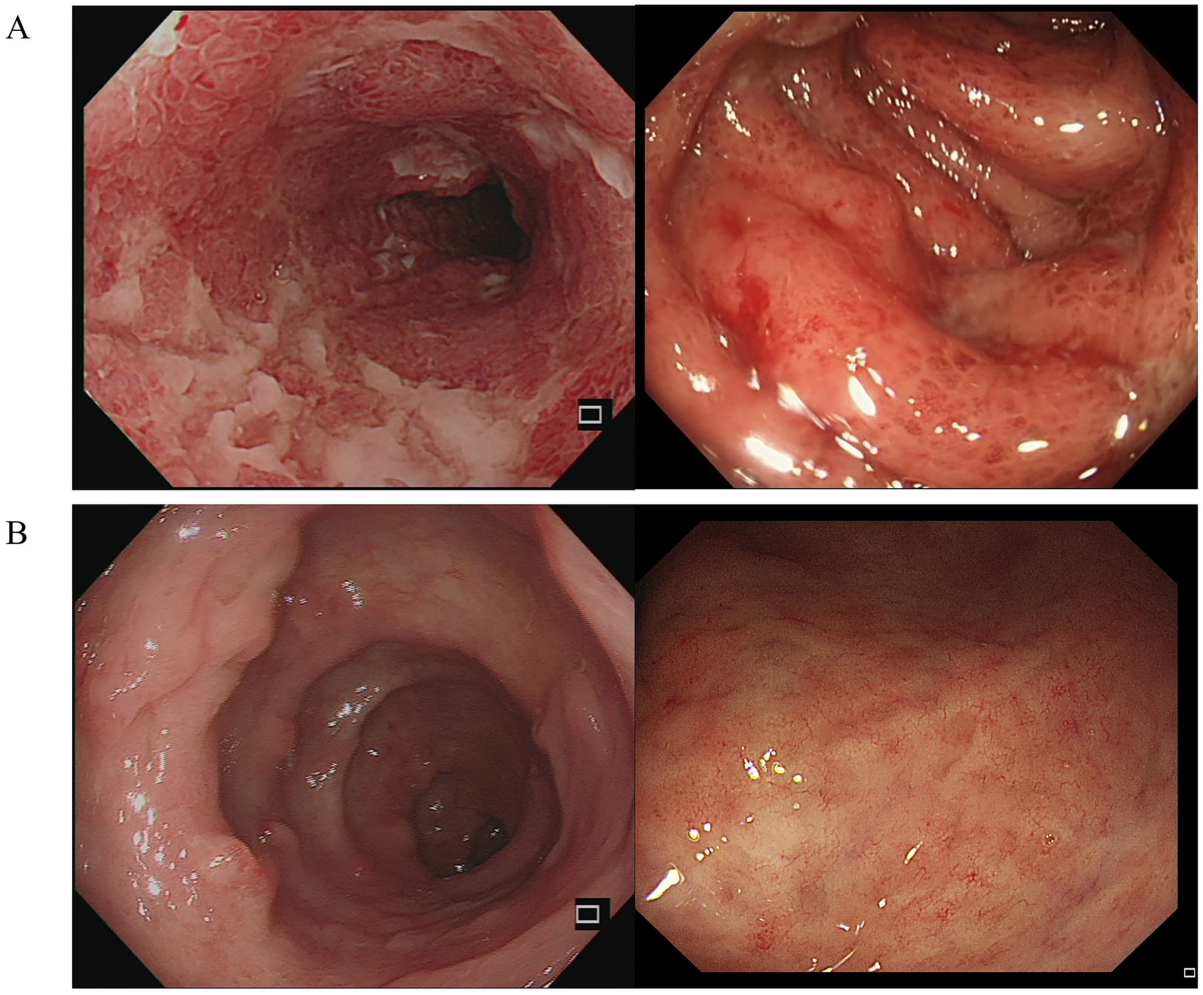
Typical endoscopic images. **(A)** The typical endoscopic images of Da-Chang-Shi-Re pattern. The intestinal mucosa exhibits marked hyperemia and edema, accompanied by ulceration; the tissue is friable and bleeds easily upon contact. **(B)** The typical endoscopic images of Pi-Xu-Shi-Yun pattern. The intestinal mucosa shows mild hyperemia and edema, accompanied by pseudopolyps and scarring.

**Figure 2:**
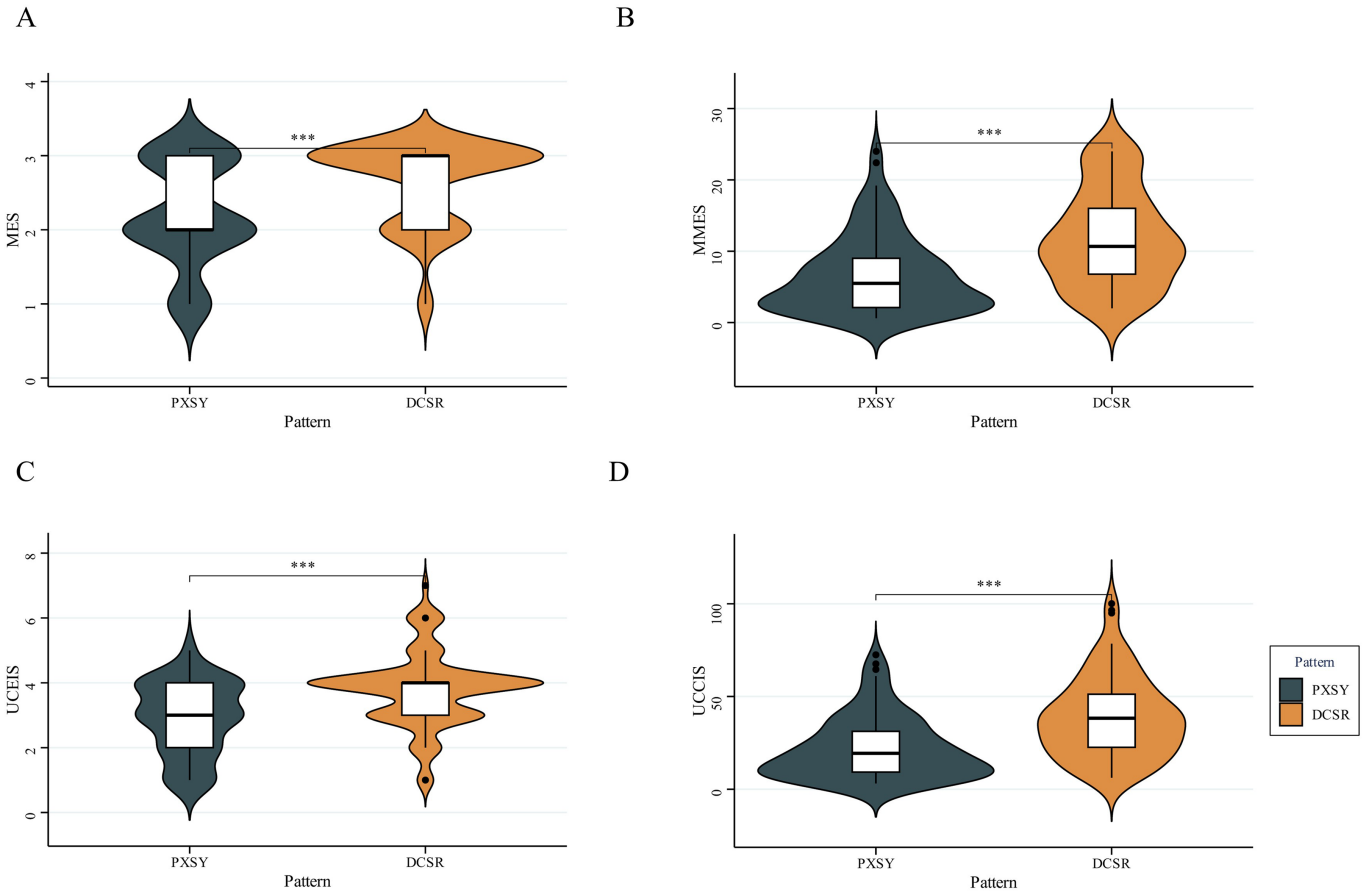
Comparison of endoscopic scores between different patterns. **(A)** Comparison of Mayo Endoscopic Score (MES) between patterns. **(B)** Comparison of Modified Mayo Endoscopic Score (MMES) between patterns. **(C)** Comparison of Ulcerative Colitis Endoscopic Index of Severity (UCEIS) between patterns. **(D)** Comparison of Ulcerative Colitis Clinical Index of Severity (UCCIS) between patterns. ns, *p* > 0.05; *p < 0.05; **, *p* < 0.01; ***, *p* < 0.001. The width of each violin plot corresponds to the density of the data distribution. Vertical lines extending from each violin shows the 95% confidence intervals. The upper and lower edges of the boxplot, respectively, denote the third quartile and first quartile. Scores on the MES, MMES, UCEIS, and UCCIS were all significantly higher in patients with DCSR pattern than in those with PXSY pattern.

We found significant differences in endoscopic manifestations between the two TCM patterns. Figure 1 presented representative colonoscopic images of the DCSR pattern (Figure 1A) and the PXSY pattern (Figure 1B). The DCSR pattern demonstrated significantly higher scores across all endoscopic evaluation systems compared with the PXSY pattern (*p* < 0.001) (Figure 2 and Table 3). The kappa values for MES (*κ* = 0.952), MMES (*κ* = 0.942), UCEIS (*κ* = 0.918) and UCCIS (*κ* = 0.937) indicate strong inter-observer agreement.

**Table 3:** Endoscopic scores and characteristics of UC participants.

Variables	DCSR (*n* = 90)	PXSY (*n* = 90)	*p*
MES, M (Q₁, Q_3_)	3.00 (2.00, 3.00)	2.00 (2.00, 3.00)	<0.001
MMES, M (Q₁, Q_3_)	10.67 (6.78, 16.00)	5.50 (2.10, 9.00)	<0.001
UCEIS, M (Q₁, Q_3_)	4.00 (3.00, 4.00)	3.00 (2.00, 4.00)	<0.001
UCCIS, M (Q₁, Q_3_)	38.30 (23.27, 51.20)	19.35 (9.25, 31.23)	<0.001
Hemorrhage, *n* (%)	44 (48.89)	21 (23.33)	0.015
Erosion, *n* (%)	80 (88.89)	73 (81.11)	0.144
Purulent discharge, *n* (%)	48 (53.33)	24 (26.67)	<0.001
Vascular changes, *n* (%)	87 (96.67)	84 (93.33)	0.494
Pseudopolyp, *n* (%)	9 (10.00)	17 (18.89)	0.090
Intestinal lumen stricture, *n* (%)	13 (14.44)	1 (1.11)	<0.001
Scar, *n* (%)	9 (10.00)	12 (13.33)	0.486
Ulcer, *n* (%)	63 (70.00)	35 (38.89)	<0.001

In terms of specific endoscopic features, patients with the DCSR pattern were more likely to present with hemorrhage, purulent discharge, ulcers, and intestinal lumen strictures (*p* < 0.001) (Table 4). These associations remained significant after adjustment for sex, medication history, extent of intestinal lesions, disease duration, and smoking status (Table 4). After adjustment for endoscopic factors in a multivariate regression analysis, ulcer formation (OR = 2.429, 95% CI: 1.209–4.880, *p* = 0.013) was still found to be independently associated with the DCSR pattern (Table 5).

**Table 4:** Univariate and multivariate logistic regression analysis of endoscopic characteristics in UC patients.

Variables	Univariate				Multivariate*				Adjusted-*p*
	*β*	S.E	*p*	OR (95% CI)	*β*	S.E	*p*	OR (95% CI)	
Hemorrhage	1.145	0.326	<0.001	3.143 (1.657 ~ 5.960)	1.127	0.359	0.002	3.086 (1.526 ~ 6.240)	0.004
Purulent discharge	1.145	0.319	<0.001	3.143 (1.683 ~ 5.868)	1.023	0.339	0.003	2.783(1.432 ~ 5.408)	0.004
Intestinal lumen stricture	2.710	1.049	0.010	15.026 (1.921 ~ 117.505)	3.352	1.192	0.005	28.561 (2.760 ~ 295.584)	0.005
Ulcer	1.299	0.316	<0.001	3.667(1.975 ~ 6.808)	1.293	0.343	<0.001	3.643 (1.860 ~ 7.132)	<0.004

*Adjusted confounders: gender, medication history, extent of intestinal lesions, disease duration, and smoking status.

**Table 5:** Multivariate logistic regression analysis of endoscopic characteristics in UC patients.

Variables	*β*	S.E	*p*	OR(95% CI)
Hemorrhage	0.351	0.394	0.373	1.421(0.656 ~ 3.078)
Purulent discharge	0.508	0.390	0.192	1.663 (0.775 ~ 3.568)
Intestinal lumen stricture	2.090	1.089	0.055	8.088 (0.956 ~ 68.406)
Ulcer	0.887	0.356	0.013	2.429 (1.209 ~ 4.880)

### 3.4 The difference of histopathological features between two TCM patterns

**Figure 3:**
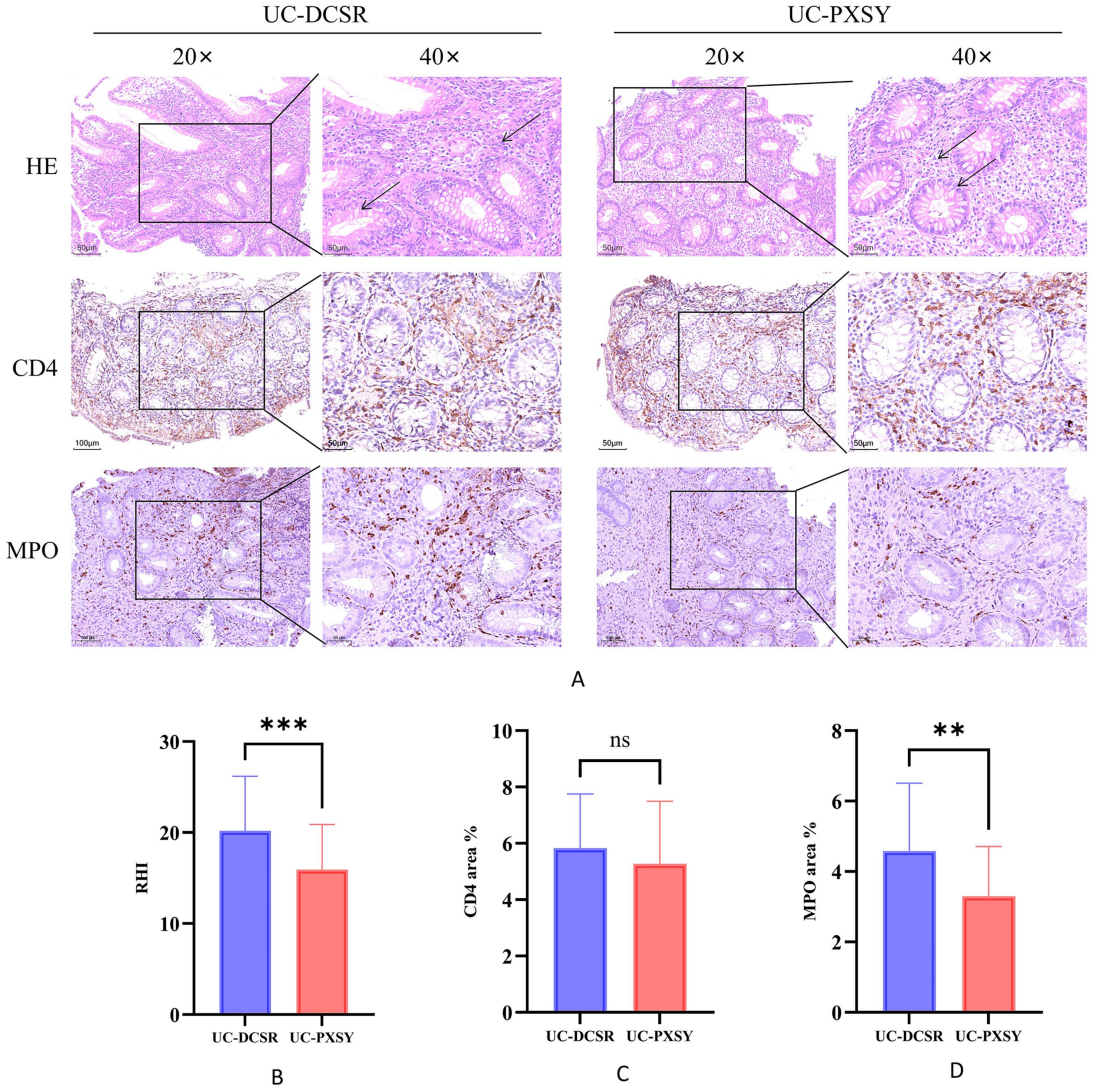
Results of histopathological analysis and immunohistochemistry between two groups. **(A)** Representative images of hematoxylin and eosin and immunohistochemistry staining. **(B)** Comparison of Robarts Histopathology Index scores between patterns. **(C)** Comparison of CD4 expression between two groups. **(D)** Comparison of myeloperoxidase expression between two groups. ns, *p* > 0.05; *p < 0.05; **p < 0.01; ***p < 0.001.

We further compared the histopathological features of the different TCM patterns. We found that the DCSR group exhibited significantly more severe mucosal damage, characterized by a reduction in goblet cells, marked disruption of crypt structures, and extensive intraepithelial inflammatory cell infiltration. Notably, extensive infiltration of NEUs were observed in the lamina propria and submucosa (Figure 3A). In contrast, the PXSY group presented with comparatively milder mucosal damage. Mucosal structures were partially preserved, with only focal epithelial damage and moderate reduction of goblet cells. Inflammatory cell infiltration was primarily confined to the lamina propria, with a reduced NEU count. The RHI score for the DCSR group was significantly higher than that for the PXSY group (*p* < 0.001), indicating greater histological activity in the DCSR pattern (Figure 3B).

Immunohistochemistry was employed to evaluate the immune cell infiltration profile in the colon tissues of patients with two distinct patterns. CD4 serves as a marker for helper T cells, while MPO indicates NEU infiltration and activation. Our findings revealed that, when compared to the PXSY group, the DCSR group demonstrated no significant difference in CD4 expression (*p* > 0.05) (Figures 3A,C). In contrast, MPO expression in the DCSR group was significantly elevated (*p* < 0.01) (Figures 3A,D). This implied that the histological differences observed between the two groups were more closely associated with NEUs rather than helper T cells. Based on this, we further examined the differences in NEU activation between the two groups.

**Figure 4:**
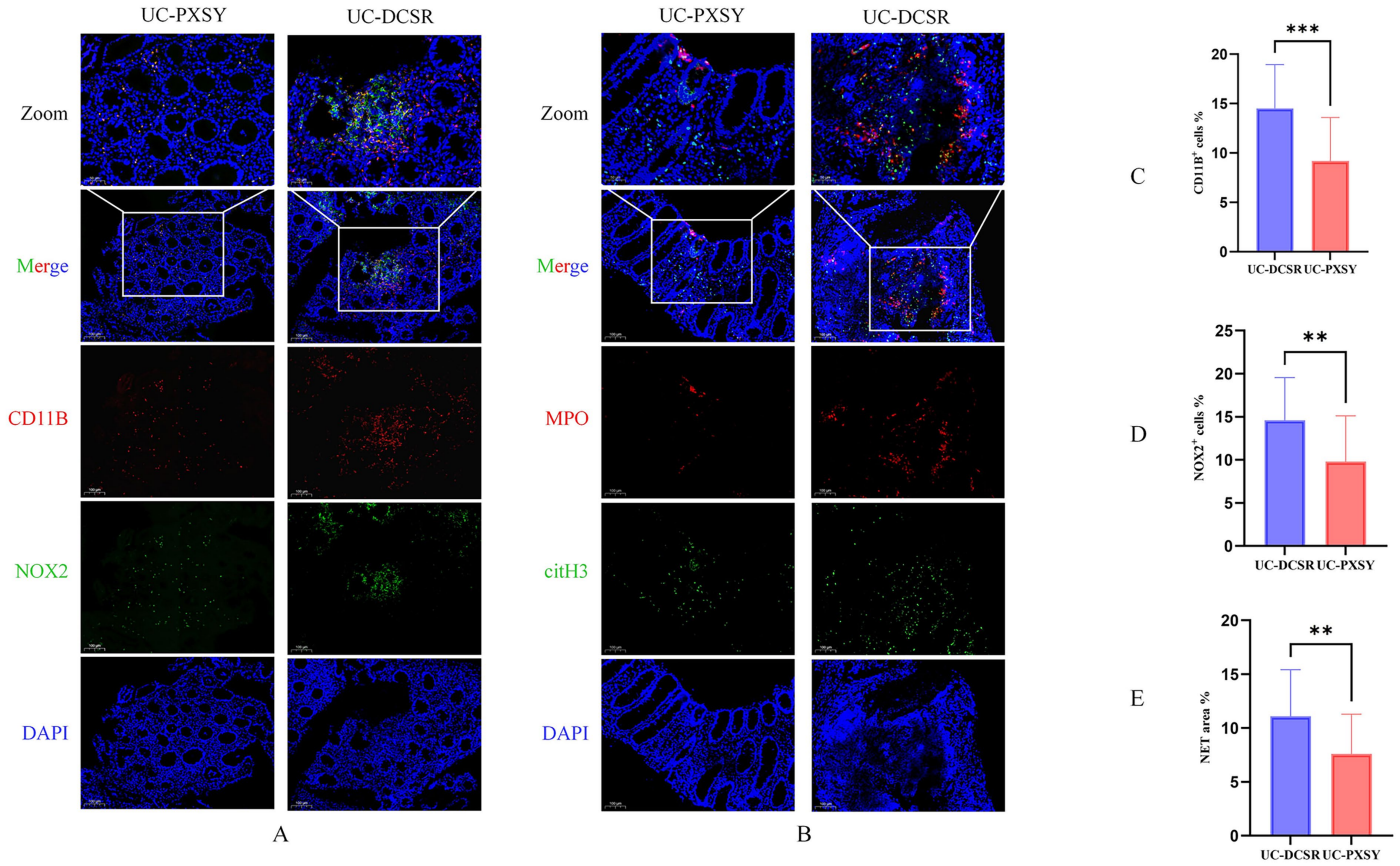
Differences in neutrophil activation between the two groups **(A)** Representative images of CD11B and NOX2 immunofluorescence. **(B)** Representative images of neutrophil extracellular traps (NET) immunofluorescence. **(C)** Quantitative comparison of CD11B expression between the two groups. **(D)** Quantitative comparison of NOX2 expression between the two groups. **(E)** Quantitative comparison of NET area between the two groups.

CD11B is a canonical surface biomarker indicating NEU recruitment and activation. NADPH oxidase 2 is responsible for reactive oxygen species production, an essential upstream event of neutrophil extracellular trap (NET) formation. Citrullinated histone H3 (citH3) is a specific marker of mature NETs. We performed immunofluorescence staining of these three indicators to evaluate intergroup differences in NEU activation (Figure 4). Compared with the PXSY group, the DCSR group exhibited stronger immunofluorescence signals for CD11B and NOX2, accompanied by a substantial increase in NET areas (Figures 4A,B). Consistently, quantitative measurements verified the upregulation of CD11B, NOX2 expression and expanded NET areas in the DCSR group (Figures 4C–E).

## 4 Discussion

This study systematically compared the differences between the DCSR and PXSY patterns in terms of laboratory indicators, endoscopic features, and histopathology. The results consistently suggested that the DCSR pattern was characterized by more severe systemic and local inflammation, while the PXSY pattern reflected a milder, more chronic inflammatory state. These findings provided strong evidence for the objectification and standardization of TCM pattern differentiation.

Many studies have shown that various laboratory markers, including CRP, ESR, ALB, and PLT, can be used to assess the severity of UC (20, 21). In recent years, novel inflammation burden markers such as NLR, PLR, NAR, and LMR have shown greater advantages in evaluating UC’s inflammatory load and predicting prognosis (14, 22, 23). We found that the DCSR pattern had higher levels of NEU, PLT, CRP, and ESR, while LYM and ALB levels were lower. Further analysis revealed that the DCSR pattern had significantly higher NLR, NAR, and PLR compared to the PXSY group. Compared to traditional indicators, these emerging biomarkers are better able to reflect the differences in peripheral inflammatory burden between the two pattern types.

Our data also demonstrated that patients presenting the DCSR endoscopic pattern exhibited substantially higher endoscopic scores relative to those with the PXSY pattern. When comparing specific endoscopic lesions, mucosal hemorrhage, ulceration, luminal stenosis and purulent discharge were all significantly more frequent in the DCSR pattern. Multivariate regression further identified mucosal ulceration independently associated to the DCSR pattern. Intestinal ulceration arises from robust inflammatory cascades driven by massive immune cell infiltration into the submucosa and muscularis propria, with NEUs serving as central effector cells in this pathological process (24, 25). Exaggerated inflammation disrupts epithelial barrier integrity, injures submucosal microvessels and ultimately causes mucosal hemorrhage (26). Collectively, these observations support that the DCSR pattern corresponds to a more severe endoscopic inflammation in UC.

At the histological level, tissues from patients with the DCSR pattern presented aggravated histological inflammation, manifested as goblet cell loss, severe distortion of crypt architecture, and extensive inflammatory cell infiltration. Immunohistochemical assays showed markedly higher MPO expression in the DCSR group, whereas CD4 expression was comparable between groups. This NEU-predominant inflammatory signature is consistent with elevated peripheral NEU counts and NEU-derived composite inflammatory indices detected in patients with the DCSR pattern. Further immunofluorescence staining targeting CD11B, NOX2 and citH3 provided functional evidence supporting robust NEU activation under this pattern. CD11B reflects NEU recruitment and activation status, NOX2 drives ROS generation to initiate NETosis, and citH3 serves as a specific biomarker of mature NETs. Quantification confirmed elevated CD11B and NOX2 signals together with enlarged NET areas in intestinal lesions of the DCSR pattern. Collectively, these layered histological and cellular findings imply that hyperactivated NEUs, rather than CD4^+^ T helper cells, may serve as key contributors to the distinct pathological phenotype characteristic of the DCSR pattern. NEU plays a critical role in anti-inflammatory and repair processes, but in UC patients, over-activated NEU exacerbates the inflammatory load through various mechanisms (27). NEU can secrete various cytokines and chemokines such as interleukin (IL)-1, IL-6, and Tumor Necrosis Factor-atease large amounts of reactive oxygen species, matrix metalloproteinases, MPO, causing cell damage and death, thereby impairing the intestinal barrier function (28, 29). Additionally, NEU can release NETs, further promoting the formation of an inflammatory cascade (30).

Overall, our study, is the first to integrate multi-dimensional data including laboratory indications, endoscopic features, and histological to explore the core differences between different TCM patterns. This study helps provide laboratory indications and endoscopic features as auxiliary tools for identifying specific TCM patterns. However, this study has several limitations. First, as a single-center study with a relatively small sample size, it primarily investigates two typical patterns of UC, which may limit the generalizability of the results. Future research should involve multi-center studies, exploring a broader range of TCM patterns in UC. Second, although we have preliminarily identified the relationship between inflammatory markers and TCM patterns, the underlying mechanisms still require further investigation. Third, as a cross-sectional study, this study lacks continuous follow-up of patients. Future longitudinal cohort studies could be designed to explore the association between different TCM pattern types and prognosis.

## 5 Conclusion

This study systematically assessed the differences between the DCSR and PXSY patterns, and found that UC patients with DCSR pattern exhibited more pronounced systematic and local inflammation, accompany with more severe NEU activation. This contributes to integrating TCM pattern differentiation with modern inflammatory biomarkers, providing valuable insights for more individualized treatment strategies in the management of UC.

## Data Availability

The original contributions presented in the study are included in the article/supplementary material, further inquiries can be directed to the corresponding author/s.
